# Tracking the 3-year trajectory of referrals to an early psychosis intervention service

**DOI:** 10.1177/10398562241251999

**Published:** 2024-05-09

**Authors:** Nirupamal Pitigala, Irene Zeng, Nishanth Narayanan, Sarah Cullum, Lillian Ng

**Affiliations:** 637385 Health New Zealand Te Whatu Ora Counties Manukau, Auckland, New Zealand; 1410 Auckland University of Technology, Auckland, New Zealand; 637385 Health New Zealand Te Whatu Ora Counties Manukau, Auckland, New Zealand; 637385 Health New Zealand Te Whatu Ora Counties Manukau, Auckland, New Zealand; 1415 The University of Auckland, Auckland, New Zealand; 10158 Counties Manukau Health, Auckland, New Zealand

**Keywords:** psychosis, early intervention, screening

## Abstract

**Aim:**

To review the baseline and clinical characteristics of patients referred to a New Zealand Early Psychosis Intervention (EPI) service across a 4-year timeframe.

**Method:**

We compared two cohorts, and identified variables associated with being accepted or declined, and reasons for decline, by an EPI service between 2013 and 2017.

**Results:**

There were 576 people with suspected psychosis referred to the EPI service for assessment: 300 (52%) were accepted, 221 (38%) declined and 55 (10%) were not processed. Reasons for being declined by EPI services were a long duration of psychosis (DUP, 48%) and no evidence of psychosis (47%). There were no significant differences between the accepted and declined group in Emergency Department presentations for self-harm or suicide attempts and acute admissions to a psychiatric inpatient unit over the 3-year follow-up period.

**Conclusion:**

To optimise the identification of true positive cases, EPI services require clear entry criteria. Replicating this study in other EPI services with different entry criteria may provide evidence to develop a more uniform screening process. Improved outcomes may be enhanced by measuring effectiveness and liaising with other EPI services.

Psychotic disorders converge with other risk factors,^1^ often at a critical developmental phase, as young adults attempt to establish their identity and achieve life tasks in education, work and relationships.^2^ Early detection and intervention are recommended^3–6^ as a longer duration of untreated psychosis (DUP) is associated with functional decline^7^ and relapse.^8^ The retrospective assessment of DUP can be challenging. The Comprehensive Assessment of At-Risk Mental State (CAARMS) is a semi-structured assessment tool used by mental health professionals to identify young people at very high risk of developing psychosis.^9^ The CAARMS can also be used to track the range and severity of symptoms over time^7^ and to identify the onset of first episode psychosis. DUP may have a different impact on various outcomes,^4,10^ in particular high use of acute mental health care,^11^ quality of life and global functioning.^12^

Meta-analytical evidence demonstrates high prospective stability in the diagnosis of psychosis.^13^ Early Psychosis Intervention (EPI) programs aim to improve symptomatic and functional recovery, to increase quality of life and satisfaction with care.^14–16^ EPI services typically require clients to meet a defined set of inclusion criteria and may impose restrictions on age, diagnosis, co-morbidities, length of illness (DUP), or prior duration of antipsychotic treatment.^17^ A definition of DUP may be from the onset of first psychotic symptoms to initiation of antipsychotic treatment.^4,8^ Early intervention services for first-episode psychosis are considered cost-effective^18–20^ and are associated with better outcomes compared with standard treatment.^21^ There is limited knowledge about the illness trajectory of those who are not accepted to EPI services beyond 3 years, the timeframe that early intervention services are usually provided.^22^ Internationally, the first study to explore ongoing mental health service use in people screened by an EPI program found that 58% of those screened were not admitted to an EPI service.^17^ Further research on the reasons for decline to EPI programs was recommended as it was cited a limitation of this study.

The criteria used for acceptance to our EPI service are a diagnosis of psychosis, age limit 16–30 years, DUP less than 2 years and past treatment with antipsychotic medication of less than 3 months. Our EPI service accepts referrals after individuals have been assessed by psychiatrists who suspect early psychosis. The EPI service conducts in-person screening to ascertain suitability of referrals. At the time of the initial intake, a comprehensive psychiatric assessment is completed. At the time of screening by the EPI service, a review and second assessment is undertaken. The screener can be of any professional discipline (EPI service psychiatrist, specialised senior medical doctor, psychologist, registrar, psychologist, registered nurse, occupational therapist and social worker). Our service does not use a standardised tool (such as the CAARMS) during screening.

In New Zealand, there has been little research conducted on baseline and clinical characteristics of patients who were referred to EPI services, screening of referrals or the trajectory of those referred. Therefore, we aimed to review the demographic and clinical profile of all individuals referred to our EPI service after an initial diagnosis of suspected early psychosis was made for those who were accepted and declined over a 3-year period. Reasons for non-acceptance to EPI services and the occupation of the EPI assessors were also reviewed. Additionally, we obtained the final diagnosis at the end of 3-year period.

## Method

This was a retrospective, observational study of an EPI service with 576 referrals in South Auckland, New Zealand, an area that covers a population of 587,650. The profiles of two cohorts (accepted or declined by the EPI service) from two electronic patient information databases over a 4-year timeframe (2013–2017) were matched. The following information was collated: referral source, age, ethnicity, gender, provisional diagnosis, substance use, the EPI screening result (accepted or declined), occupation of the EPI assessor, reasons for decline and baseline clinical variables available prior to EPI referral, the number of acute Emergency Department (ED) presentations with self-harm and/or suicide attempts, admission(s) to a psychiatric inpatient unit, use of mental health legislation, risks and update in diagnosis over a 3-year period.

Information was obtained from electronic health records for people not accepted to EPI services who remained in the hospital catchment area, were referred to community health services, re-presented to services via the ED or were re-referred. Socio-demographic data, clinical characteristics, reasons for decline to the EPI service and illness trajectories of service users between the two cohorts were compared.

Descriptive statistics were used to ascertain the declined referral rate to the EPI service during the study period according to demographics, initial assessors and reasons for decline to EPI. We applied logistic regression analysis to identify significant variables (demographics and clinical factors) associated with being accepted or declined to the EPI service. Survival analysis (Cox regression) was used to compare the rates of acute admissions to a psychiatric unit and ED admissions between the two cohorts accounting for the time-to-event variation, adjusted for other covariates including those identified from the abovementioned multiple logistic regression. Covariates in Cox regressions included age-at-time of referral, year of referral, ethnicity, gender, previous ED admission due to self-harm/suicide attempts within 6 months prior to referral, inpatient acute mental admission within 6 months prior to referral and substance use within 3-years prior to referral. ED and MH Act related first readmission outcomes analyses were stratified by ethnicity, as the sample had a high proportion of Māori and Pasifika, who are known to have higher rates of compulsory mental health treatment over time.^23^ Life test and Kaplan–Meir curves were calculated for acute ED admissions with self-harm or suicide attempts and acute psychiatric inpatient admissions within the 3-year follow up period, stratified by EPI referral assessment outcome. Propensity score for cohort matching was derived based on variables: age at time of referral, year, ethnicity, gender, ED and previous psychiatric admissions within 6 months ^17^. Due to a relatively small number of both cohorts, these matching variables were included in the multi-variable analyses. The study was approved by the Auckland Health Research Ethics Committee.

## Results

The study cohort included 576 EPI service referrals, of which 300 (52%) referrals were accepted, 221 (38%) were declined and 55 (10%) were not processed. In the declined group, 72/221 (12%) were declined without being screened, compared to 100% of the accepted group who were screened. In this section, we highlight demographic and clinical information for the accepted and declined cohorts (Table 1), reasons for decline by the EPI service, self-harm and suicide attempts within the 3-year follow up (Table 2) and rates of subsequent inpatient admission.

**Table 1.** Baseline demographics and clinical characteristics

Demographics and clinical characteristics		EPI service assessment outcome				
Referrals to EPI service		Screened		Unscreened		*p* value
		Accepted	Declined	Declined	Unrecorded	
Gender	Female	83 (27.67%)	52 (35.14%)	22 (30.56%)	22 (40%)	
	Male	217 (72.33%)	96 (64.86%)	50 (69.44%)	33 (60%)	
	Total	300	148	72	55	.19
Ethnicity	Asian	47 (15.67%)	20 (13.51%)	6 (8.33%)	10 (18.18%)	
	Māori	94 (31.33%)	47 (31.76%)	28 (38.89%)	20 (36.36%)	
	European & other	76 (25.33%)	50 (33.78%)	20 (27.78%)	14 (25.45%)	
	Pacific	83 (27.67%)	31 (20.95%)	18 (25%)	11 (20%)	
	Total	300	148	72	55	.43
Substance use 3 years prior to EPI service referral	No	92 (30.87%)	54 (36.73%)	27 (40.3%)	6 (15.79%)	
	Yes	206 (69.13%)	93 (63.27%)	40 (59.7%)	32 (84.21%)	
	Total	298	147	67	38	.04
Antipsychotics used 3 years prior to EPI service referral	No	91 (30.54%)	65 (44.22%)	19 (28.36%)	11 (28.21%)	
	Yes	207 (69.46%)	82 (55.78%)	48 (71.64%)	28 (71.79%)	
	Total	298	147	67	39	.019
Contact with Forensics 3 years prior to EPI service referral	No	273 (91.3%)	136 (92.52%)	61 (91.04%	34 (87.18%)	
	Yes	26 (8.7%)	11 (7.48%)	6 (8.96%)	5 (12.82&)	
	Total	299	147	67	39	.77
Number of MHA used 3 years prior to EPI service referral	0	209 (70.61%)	109 (74.66%)	48 (73.85%)	24 (61.54%)	
	1	84 (28.38%	36 (24.66%)	16 (24.62%)	15 (38.46%)	
	≥2	3 (1.01%)	1 (0.68%)	1 (1.54%)	0 (0%)	
	Total	296	146	65	39	.6

**Table 2.** First ED admission and EPI service assessment outcome^a^

Parameter		Hazard ratio	95% confidence interval of hazard ratio	*p* values
EPI service assessment outcome	Declined vs accepted	0.9	0.53–1.51	.68
	Undocumented vs accepted	0.58	0.18–1.91	.37
Age at time of referral		0.94	0.87–1.02	.14
Year of referral		1.2	0.98–1.46	.08
Gender	Female vs male	1.46	0.87–2.42	.15
ED admission due to self-harm or suicide attempts 6 months prior to referral	Yes vs No	2.34	1.09–5.04	.03^a^
Inpatient admission 6 months prior to referral	Yes vs No	1.64	0.98–2.76	.06
Substance use 3 years prior to referral	Yes vs No	1.19	0.67–2.11	.56

^a^Adjusted by known confounding factors.

### Sociodemographic and clinical characteristics prior to referral

There was no significant difference in gender, ethnicity and clinical characteristics among the cohort who were accepted, declined, declined without being screened or not processed except for their substance use history. Out of 221 people who were declined to EPI service, 88% were recorded as referred to community mental health service during the follow-up period.

The main reasons for patients being declined by EPI were a long DUP (48%) and no evidence of psychosis (47%). The actual DUP in each group was not specified in the health records. Of the 70 participants who were declined due to no evidence of psychosis at the time of the EPI service referral, 13 were diagnosed with psychosis 3-years later (18.6%). The occupation of the assessors who declined referrals due to the reason ‘no evidence of psychosis’ were mostly senior doctors not vocationally trained in psychiatry (20.4%), followed by psychiatrists (16.3%), psychologists (15.3%) and nurses/registrars (6.1%).

Factors associated with likelihood of EPI service acceptance were age and ethnicity at the time of referral. Young people <20 years were more likely to be accepted to EPI services than those aged ≥20, and people of Pacific ethnicity were more likely to be accepted than other ethnicities.

### Outcomes at three-year follow-up

There was no significant difference in the number of self-harm and suicide attempts within the 3-year follow-up period between the accepted and declined cohorts (Table 2). The results were the same after adjusting for other risk factors. Prior ED admission due to self-harm or suicide attempts 6 months within an EPI referral was a significant factor associated with an ED admission in the follow-up (hazard ratio: 2.34 (95% CI:1.09, 5.04), *p* = .03). Notably, there were two deaths by suicide in the accepted group and three in the declined group.

Previous inpatient admission 6 month within referral was significantly associated with a psychiatric inpatient unit admission during the follow up (hazard ratio: 2.60 (95% CI: 1.89–3.57), *p* < .0001, Table 3).

**Table 3.** First inpatient mental health admission and EPI service assessment outcome^a^

Parameter		Hazard ratio	95% confidence interval of hazard ratio	*p* values
EPI service assessment outcome	Declined vs accepted	0.82	0.6–1.14	.24
	Unrecorded vs accepted	0.56	0.28–1.11	.10
Age at time of referral		0.99	0.95–1.04	.80
Year of referral		0.99	0.89–1.10	.89
Ethnicity	Asian vs. European/other	0.77	0.44–1.35	.36
Ethnicity	Māori vs. European/other	1.02	0.69–1.52	.92
Ethnicity	Pacific vs. European/other	1.21	0.81–1.82	.36
Gender	Female vs male	0.85	0.61–1.20	.36
ED admission due to self-harm or suicide attempts 6 months prior to referral	Yes vs no	0.91	0.47–1.74	.77
Inpatient admission 6 month prior to referral	Yes vs no	2.60	1.89–3.57	<.0001

^a^Adjusted by known confounding factors.

## Discussion

We highlight three main findings from this study. First, the reasons for decline to this EPI service were most commonly a long DUP and ‘no evidence of psychosis’. Second, there was no significant difference in outcome variables between the two groups. The third finding relates to the proportion of referrals that were not screened.

Eighteen percent of those who were declined due to no evidence of psychosis were later diagnosed with a schizophrenia spectrum disorder, which raises the possibility of missing true positives at the time of screening by the EPI service. A challenge in first episode psychosis is that a diagnosis at the time of referral to EPI services is often not clearcut. Intervention in the prodromal phase may delay or prevent more severe illness but raises the potential for false positives, that is, people at risk who do not go on to develop the disorder.^24^ There is meta-analytic evidence for a high stability of a diagnosis of first-episode psychosis, from the time of diagnosis.^13^ Clinicians trained in the use of a standardised tool, such as the CAARMS, in screening may more accurately ascertain the DUP and diagnosis. The above challenges could be managed by ongoing training for EPI service screeners, including the CAARMS in the screening process, reviewing entry criteria to EPI services (e.g. the length of DUP), regular auditing of the declined and not-seen groups to improve the screening process and obtaining a second opinion for presentations that are diagnostically challenging.^9^ There are variable definitions of DUP and it would be beneficial for EPI services to have entry criteria that specify a standardised cut-off length of DUP.^12^

The second main finding of this study is that there were no significant differences, in terms of outcome variables, between the two groups. The declined and accepted groups had comparable outcomes, which may reflect adequate standard case management. Despite evidence that the implementation of EPI services is cost-effective across different health systems,^18–20^ there is debate about early intervention compared with standard care for the treatment of first-episode psychosis. In a general mental health context, aspects of an EPI service model^26,27^ may be used. Generalists can use a clinical staging approach and broad spectrum simpler treatments for less well differentiated early phases of psychosis.^25^ The effectiveness of EPI services may be measured by key performance indicators with clinical governance oversight.^28^ Liaising with other EPI services, nationally and internationally may enable comparison of outcomes. More research is required to rigorously assess the value of EPI services before expansion, particularly in a time when mental health budgets are more constrained.

Finally, referrals that are not screened (including those declined without being screened) are a major concern. Those who are not screened by an EPI service require active follow up and engagement to ensure they receive appropriate treatment.

## Strengths and limitations

The 3-year follow up duration, the large number of participants, and the inclusion of reasons for patients being declined to an EPI service are strengths of this study. The sample is limited in that the two cohorts were not evenly matched, with variables (from literature and the data) used as confounding effects in the comparison. We acknowledge the limitations of missing data: those discharged to primary care or to other mental health services in another locality, and those referred to EPI services for whom we could not find any documentation. Future research might include a comparison of outcomes based on inclusion criteria between EPI services, a prospective assessment of functional outcomes and qualitative methods to understand lived experience and engagement with EPI services. Replication of this study in other EPI services would provide more information about how the EPI model is used in screening and case management.

## Conclusion

Evaluating DUP and diagnosing early psychosis require high competence in clinical skills and applying standardised tools, such as the CAARMS. EPI services require training of skilled assessors to utilise the CAARMS. To optimise the identification of true positive cases, EPI services require clear entry criteria, including a specified DUP. Replicating this study in other EPI services with different entry criteria may provide evidence to develop a more uniform screening process. EPI services require key performance indicators to measure their effectiveness and monitoring through clinical governance. Liaising with other EPI services nationally and internationally may improve outcomes for service users.

## Data Availability

The authors report direct access to the study data, which have been stored in accordance with Auckland Health Research Ethics Committee guidelines.*
